# Identification and Characterization of Neuroprotective Properties of Thaumatin-like Protein 1a from *Annurca* Apple Flesh Polyphenol Extract

**DOI:** 10.3390/nu16020307

**Published:** 2024-01-19

**Authors:** Antonio D’Errico, Rosarita Nasso, Antimo Di Maro, Nicola Landi, Angela Chambery, Rosita Russo, Stefania D’Angelo, Mariorosario Masullo, Rosaria Arcone

**Affiliations:** 1 Department of Medical, Movement and Well-Being Sciences (DiSMMeB), University of Naples “Parthenope”, Via Medina 40, 80133 Napoli, Italy; antonio.derrico002@studenti.uniparthenope.it (A.D.); rosaritanasso@gmail.com (R.N.); stefania.dangelo@uniparthenope.it (S.D.); mario.masullo@uniparthenope.it (M.M.); 2 Department of Environmental, Biological and Pharmaceutical Sciences and Technologies (DiSTABiF), University of Campania “Luigi Vanvitelli”, Via Vivaldi 43, 81100 Caserta, Italy; antimo.dimaro@unicampania.it (A.D.M.); nicola.landi@unicampania.it (N.L.); angela.chambery@unicampania.it (A.C.); rosita.russo@unicampania.it (R.R.); 3 Institute of Crystallography, National Research Council of Italy, Via Vivaldi 43, 81100 Caserta, Italy

**Keywords:** thaumatin-like protein 1a, *Annurca* apple polyphenols, cholinesterase inhibitor, monoamine oxidase (MAO) inhibitor, A*β*_1–40_ aggregation, Alzheimer’s disease (AD), Parkinson’s disease (PD)

## Abstract

Background: Alzheimer’s disease (AD) and Parkinson’s disease (PD) are multifactorial neurodegenerative disorders that are mostly treated with drugs inhibiting key enzymes of cholinergic and aminergic neurotransmission, such as acetyl and butyryl cholinesterase (AChE, BuChE) or monoamine oxidases (MAO)-A/B, and of A*β* _1–40_ aggregation. Diet plant components with multitarget functions are promising compounds in the prevention of AD and PD. Our aim was to identify neuroprotective compounds from *Annurca* apple polyphenol extract (AFPE). Methods: AFPE was fractionated by gel filtration, and the eluted peaks were subjected to chemical analyses (i.e., RP-HPLC and mass spectrometry), determination of inhibitory enzyme activity and cell effects by MTT, and morphology assays. Results: In AFPE, we identified thaumatin-like protein 1a, belonging to the pathogenesis-related protein (PR) family. This protein showed the best inhibitory activity on AChE, MAO-A (IC_50_ = 5.53 µM and 1.71 µM, respectively), and A*β*_1–40_ fibril aggregation (IC_50_ = 9.16 µM), compared to AFPE and other polyphenol-containing fractions. Among the latter, Peak 4 reverted A*β* fibril formation (IC_50_ = 104.87 µM). Moreover, thaumatin-like protein 1a protected AGS and MKN-28 cells from serum-deprivation-induced stress conditions. Conclusions: We showed that AFPE exerted neuroprotective functions not only through its polyphenols but also through thaumatin-like protein 1a, which acted like a multitarget molecule.

## 1. Introduction

Among neurodegenerative disorders, Alzheimer’s disease (AD) and Parkinson’s disease (PD) are the most widespread pathologies of the central nervous system (CNS) that affect elderly people [1]. AD and PD are multifactorial disorders leading to a progressive and irreversible death of cholinergic neurons, which causes severe cognitive and motor dysfunctions. AD and PD share some similarities at the cellular and molecular levels because their hallmarks are represented by the accumulation of protein aggregates in the patient’s brain (amyloid beta (A*β*) and tau protein in AD, and alpha-synuclein in PD) that cause neuronal death [2]. Neuronal death is accompanied by decreases in cholinergic and aminergic neurotransmitters; hence, the main therapeutic approaches aim to restore these neurotransmitters’ levels through the administration of specific inhibitors of key enzymes involved in their metabolism, as well as NMDA receptor antagonists, such as rivastigmine, galantamine, and donepezil [3].

However, these drugs can ameliorate cognitive and motor dysfunctions without reverting the cause of the disorders, and they can cause various side effects; therefore, a promising strategy is represented by natural and diet plant compounds, either as lead structural compounds to develop new drugs or as molecules to be used against neurodegenerative disorders [3,4,5,6].

In recent years, many efforts have been undertaken to identify diet compounds that can act as multifactorial agents capable of preventing, delaying, and counteracting the onset of AD and PD [6,7]. The Mediterranean diet contributes to a healthy status, and it also exerts anti-inflammatory, chemopreventive, and neuroprotective effects that are mostly due to its high intake of antioxidants and polyphenols, which are very abundant in legumes, vegetables, and fruits [8,9,10,11,12]. The apple (*Malus domestica*), one of the most consumed fruits in the Mediterranean area, is rich in fibers, vitamins (vitC), minerals such as potassium, and various antioxidant components, mostly polyphenols that are known for their beneficial effects on human health [13]. However, among apple flesh’s components, the presence of proteins with structural end enzymatic functions has been reported, and their biological properties are still under investigation [14,15].

In apples, which are very widespread in Southern Italy, the presence of thaumatin-like protein 1 (TLP-1/s) has been already reported, and its characteristics can vary depending on various factors, such as the apple variety, growing conditions, and ripening [16,17,18,19]. TLPs have been found in various fruits, such as apples, cherries, bananas, and kiwis [20]. TLPs consist of 21–26 kDa polypeptides sharing a 40–70% sequence homology to thaumatin, a super-sweet 22 kDa polypeptide isolated from the African plant katemfe (*Thaumatococcus daniellii*) [21]. For its properties, thaumatin was approved as a safe low-calorie sweetener to be used in the food and pharmaceutical industries in various countries [22,23].

TLPs are mostly related to plants’ defense mechanisms against pathogens, insects, environmental stresses, and food [16,20]. TLPs are classified as part of the pathogenesis-related protein-5 (PR-5) family, to which many proteins sharing significant sequence similarity and functions belong [16,20]. PR-5 expression is induced by different phytopathogens and stress conditions in many plants, including apples, and their roles are related to the plant’s defense against pathogens, but they are also important for fruit allergy research [15,20,24].

Among the plant proteins belonging to the PR-5 family, osmotin, a 26 kDa protein isolated from *Nicotiana tabacum*, acts as a mammalian adiponectin homolog [25,26], showing anti-inflammatory, anti-apoptotic, and neuroprotective effects in AD and PD [27,28,29,30].

In our previous studies, we characterized the protective effects of polyphenol extracts from *Annurca* apple flesh (AFPE) [31,32] and *Citrus limon* peels [8]. These extracts exerted neuroprotective, chemopreventive, and anti-inflammatory functions, as demonstrated by studies performed using in vitro enzyme assays and in human primary colon cancer cells [8,12]. In fact, these extracts inhibited key enzymes of AD and PD involved in cholinergic metabolism, such as acetyl- and butyryl-cholinesterase (AChE and BuChE, respectively), and aminergic neurotransmission, such as monoamine oxidases (MAO)-A/B, while also reducing A*β* _1–40_ aggregation [8,32,33].

With all these findings taken together, the aims of this study were to investigate the presence of protein components and to identify some of the polyphenol constituents in AFPE that could be involved in neuroprotective effects.

To achieve these objectives, we used two main experimental approaches: first, a biochemical procedure allowing for the separation and characterization of the protein and polyphenol fractions of AFPE, and second, their functional characterization by in vitro assays to determine their inhibitory activity on cholinesterases (AChE and BuChE), monoamine oxidases (MAO-A and MAO-B), and the A*β* _1–40_ fibril aggregation process. Finally, the cytotoxic effects of the purified AFPE protein and polyphenol fractions were evaluated using cell culture models, consisting of the AGS and MKN-28 cell lines, mimicking the cell types of the gastrointestinal system involved in the metabolism of food nutrients, and the human neuroblastoma SH-SY5Y cell line, a suitable model for brain function studies.

## 2. Materials and Methods

### 2.1. Materials

AChE from *Electrophorus electricus*, BuChE from equine serum, human MAO-A and -B, acetylthiocholine, butyrylthiocholine, kynuramine, 5′,5′-dithiobis-2-nitrobenzoic acid (DTNB), thioflavin T, donepezil, clorgyline, and selegiline were purchased from Sigma-Aldrich (Milano, Italy). Human *β*-amyloid peptide (1–40, cat. ab120479) was obtained from Abcam (Cambridge, UK). Dulbecco’s modified Eagle medium (DMEM), fetal bovine serum (FBS), trypsin–EDTA, and phosphate-buffered saline (PBS) pH 7.4 were obtained from Lonza (Basel, Switzerland); 3-(4,5-dimethyl-2-thiazolyl)-2,5-diphenyl-2H-tetrazolium bromide and MTT were obtained from Sigma-Aldrich (Milano, Italy).

### 2.2. Annurca Apple Flesh Polyphenol Extraction and Polyphenol Content Evaluation

The extraction and evaluation of polyphenols from *Annurca* apple flesh were performed as previously reported [32]. Briefly, *Annurca* apple (Malus pumila cv. *Annurca*) fruits (each weighing about 100 g) were obtained from farms in Giugliano (Napoli, Italy) in October, right after the fruits were harvested (green peel). The fruits were reddened in the “melai” according to a specific procedure and then sent to the market. Polyphenol extraction from *Annurca* apple flesh was carried out using 40 g of apple flesh, as previously reported [32], using 40 mL of 80% methanol and 20% water containing 0.18 M HCl. The total polyphenolic contents in the 65 mL extract obtained were assessed using the Folin–Ciocâlteu colorimetric method [34] on an appropriate volume of the samples, and the absorbance readings were compared to a standard curve of gallic acid solutions [35]. The concentration of the extract was brought to a 10 mM gallic acid equivalent in PBS, corresponding to 2.3 g of *Annurca* Apple flesh per mL of extract. The AFPE was aliquoted and stored at –80 °C until use; (+)-catechin, (−)-epicatechin, and chlorogenic acid were the main *o*-diphenol components of AFPE identified by HPLC [32].

### 2.3. Purification and Molecular Characterization of P33 Protein from AFPE

#### 2.3.1. Desalting of P33 Protein by RP-HPLC

Desalted protein for mass spectrometry identification was obtained using a linear gradient of 0.1% TFA (solvent A) and acetonitrile containing 0.1% TFA (solvent B) from 5 to 65% of solvent B over 60 min, at a flow rate of 1.0 mL/min, on a C-4 column (250 mm × 4.6 mm, 5 μm particle size; 300 Å; Phenomenex, Castel Maggiore, Bologna, Italy). Elution was monitored at 214 nm.

#### 2.3.2. High-Resolution Nano-LC–Tandem Mass Spectrometry

Mass spectrometry analysis was carried out on the polyphenol fractions via RP-HPLC (Peak 8, 500 fmol), following trypsin digestion performed as previously described [36] by using a Q Exactive Orbitrap mass spectrometer equipped with an EASY-Spray nano-electrospray ion source (Thermo Fisher Scientific, Bremen, Germany) and coupled with a Thermo Scientific Dionex UltiMate 3000 RSLCnano system (Thermo Fisher Scientific). For data processing, the acquired raw files were analyzed with Proteome Discoverer 2.1 software (Thermo Fisher Scientific, Rockford, IL, USA) using the SEQUEST HT search engine. The HCD MS/MS spectra were searched against the UniProt_SwissProt database, taxonomy Viridiplantae (ID: 33090), assuming trypsin (Full) as a digestion enzyme, with two missed cleavage sites allowed. The mass tolerances were set to 10 ppm and 0.02 Da for the precursor and fragment ions, respectively. Oxidation of methionine (+15.995 Da) was set as the dynamic modification, and carbamidomethylation of cysteine (+57.021 Da) was set as the static modification. The false discovery rates (FDRs) for peptide-spectral matches (PSMs) were calculated and filtered using the Target Decoy PSM Validator Node in Proteome Discoverer. The Target Decoy PSM Validator Node specifies the PSM confidences based on dynamic score-based thresholds. It calculates the node-dependent score thresholds needed to determine the FDRs, which are provided as input parameters of the node. The Target Decoy PSM Validator was run with the following settings: maximum delta Cn of 0.05, a strict target FDR of 0.01, a relaxed target FDR of 0.05, and validation based on q-values. The Protein FDR Validator Node in Proteome Discoverer was used to classify protein identifications based on q-values. Proteins with a q-value < 0.01 were classified as high-confidence identifications, while proteins with a q-value of 0.01–0.05 were classified as medium-confidence identifications. Only proteins identified with medium or high confidence were retained, resulting in an overall FDR of 5%. Multiple alignment of thaumatin sequences was performed with the BLAST program, available online (http://www.ncbi.nlm.nih.gov/BLAST, accessed on 30 November 2023).

#### 2.3.3. Separation of AFPE Components by Gel-Filtration Chromatography

AFPE (1 mL) was loaded onto a HiLoad Superdex 75™ 16/60 column (Agilent Technology, Milan, Italy) connected to an FPLC system (Agilent Technology, Milan, Italy). The column was eluted and equilibrated at 1 mL/min with 20 mM Tris•HCl buffer, pH 7.8, containing 100 mM NaCl. The column was calibrated with ovoalbumin (46 kDa), carbonic anhydrase (29 kDa), and cytochrome c (12.4 kDa) as protein molecular weight standards. Material retained on the column was eluted by injecting a 1 M NaOH (1 mL) solution. The absorbance at 280 nm was continuously monitored, and fractions of 2.0 mL were collected and pooled according to their elution profile.

#### 2.3.4. Qualitative Analysis of Extracted Polyphenols by RP-HPLC Analysis

The polyphenol components of the AFPE (~40 nmol) were analyzed by RP-HPLC using a Kromasil C18 column (150 × 4.6 mm, 5 μm particle size), as previously reported [35]. The following solvents were used: solvent A (Milli-Q water containing 0.2% acetic acid) and solvent B (methanol). The column was eluted at flow rate of 1 mL/min using the following gradient: 5% B for 5 min, 15% B for 1 min, 25% B for 20 min, 100% B for 15 min, and 100% B for 4 min. The main polyphenols were identified based on the retention times of authentic standard references, monitoring the absorbance at 278 nm. The standard references used were gallic acid, (+)-catechin, chlorogenic acid, caffeic acid, (–)-epicatechin, coumaric acid, and quercetin. The injected volume for both standards and samples was 1.0 mL dissolved in solvent A.

#### 2.3.5. SDS-PAGE Protein Analysis

Protein concentration was determined by a colorimetric assay [37], and adequate amounts of sample (5–20 μg) were heated at 95 °C for 5 min in Laemmli denaturing buffer and then loaded onto 12% reducing SDS-PAGE [38]. Total protein detection was performed by Coomassie R-250 blue staining of the gel.

## 3. Enzymatic Assays

### 3.1. Cholinesterase Assay

AChE and BuChE activity was assayed by the Ellman method [39], as previously reported [5,8], using acetylthiocholine and butyrylthiocholine as substrates, respectively. Donepezil was used as an internal control for the inhibition observed. The hydrolysis of thiolated substrates was followed colorimetrically (412 nm) at room temperature (22–27 °C) using a Cary 100 UV–VIS Spectrophotometer (Agilent, Santa Clara, CA, USA). The reaction mixture (500 μL) contained 330 μM 5,5′-dithio-bis-2-nitrobenzoic acid (DTNB), 500 μM acetylthiocholine or butyrylthiocholine as a substrate, and increasing concentrations of Peak 1 or polyphenols (Peaks 2, 3, and 4) in 0.1 M sodium phosphate buffer (pH 7.4). The reaction was started by the addition of 100 mU/mL AChE or BuChE, and the initial rate of the reaction was derived from the linear portion of the kinetics. The concentration of the inhibitor required to reduce the enzymatic activity to 50% (IC_50_) was derived from semi-logarithmic plots. Linear curve fits were obtained using the least-squares method, and the significance of the correlation was estimated from the squared correlation coefficient *r*^2^.

### 3.2. Monoamine Oxidase Assay

Monoamine oxidase activity was assayed using the fluorimetric method previously reported in [32,40]. This method was based on the oxidation of kynuramine by monoamine oxidase, leading to the production of 8-hydroxychinoline, which becomes fluorescent in alkaline conditions. The 250 μL reaction mixture was prepared in a 50 mM potassium phosphate buffer, pH 7.1, and contained 40 μM kynuramine in the absence or presence of different concentrations of the components of fractioned AFPE. The reaction was started by adding monoamine oxidase A or B (3.75 μg) and allowed to proceed for 20 min. The enzymatic oxidation of the substrate was stopped by adding 150 μL of 2 M NaOH and, after 10 min of incubation at room temperature, 240 μL of water. The resulting mixture was centrifuged for 10 min at 15,000 rpm, and the fluorescence was measured on 500 μL of the supernatant using a Cary Eclipse Spectrofluorometer (Agilent Technology, Milan, Italy). The fluorescence signal was recorded at room temperature (20–25 °C) using excitation and emission wavelengths of 315 and 380 nm, respectively; the slits were set to 10 nm for both the excitation and emission beams. The residual activity was compared with that measured in the absence of the components of fractioned AFPE, and the data were collected in at least three different experiments. clorgyline and selegiline were used as positive controls for MAO-A and MAO-B, respectively, as previously reported [32]. The concentration leading to 50% residual activity (IC_50_) was derived from a semi-logarithmic plot in which the logarithm of the residual activity was plotted against the inhibitor concentration.

## 4. A*β* Self-Aggregation Assay

### 4.1. Inhibition of Aβ _1–40_ Self-Aggregation

Inhibition of A*β* _1–40_ self-aggregation was performed by incubating 96 µM peptide in 12 µL of 200 mM sodium phosphate buffer (pH 8.0) containing 0.5% (*v*/*v*) DMSO at 37 °C for 24 h, in the absence or in the presence of increasing concentrations of AFPE or Peaks 1–4, as previously reported [5]. To quantify amyloid fibrils’ formation, 0.5 mL of 1.6 µM thioflavin T in 50 mM glycine–NaOH buffer (pH 8.5) was added. Therefore, a 300 s time scan of fluorescence intensity was measured using excitation and emission wavelengths of 446 and 490 nm, respectively (the slits were set to 10 nm for both the excitation and emission beams); the fluorescence values at plateau were averaged over at least 2 min of scanning. The percentage inhibition due to the presence of the self-aggregation inhibition was calculated from the decrease in the fluorescence signal after the subtraction of the background fluorescence of a thioflavin T solution obtained in the same way. The concentration leading to 50% residual self-aggregation (IC_50_) was derived from a semi-logarithmic plot in which the logarithm of the residual self-aggregation was plotted against the inhibitor concentration.

### 4.2. Reversion of Pre-Aggregated Aβ _1–40_ Peptides

Peptide aggregation was induced as described above; then, AFPE or the single FPLC pool (Peaks 1, 2, 3, and 4) was added, and incubation was prolonged for additional 24 h. A*β*_1–40_ aggregation was measured by a thioflavin T [41] (Th-T) binding assay at the beginning of incubation, immediately before treatment with Peak 1 or 2, and 24 h after the addition of each sample.

## 5. Cell Cultures and Treatments

The human gastric adenocarcinoma MKN-28 and AGS cell lines (American Type Culture Collection, Manassas. VA, USA, ATTC CCL-107) [12], along with the neuroblastoma SH-SY5Y cell line (American Type Culture Collection, ATTC CRL-2266), were cultured in Dulbecco’s modified Eagle medium (DMEM), supplemented with 10% heat-inactivated fetal bovine serum (FBS) (Invitrogen, Life Technologies, Monza, Italy), 1.5 mM L-glutamine, 100 units/mL penicillin, and 100 μg/mL streptomycin, under a humidified atmosphere of 5% CO_2_ at 37 °C. Subconfluent cells were plated (1 × 10^4^ cell/well) onto a 96-well plate and treated with increasing concentrations of AFPE or Peaks 1–4 for 24 h in serum-free medium.

### Cell Viability Assay and Morphological Analysis

Cell viability was evaluated as mitochondrial metabolic activity using the MTT assay, as previously reported [42]. Briefly, the cells were plated onto 96-well plates (1 × 10^4^ cells/well) in DMEM with 10% FBS and, after 24 h of seeding, were treated with different concentrations of AFPE or Peaks 1–4, in serum-free medium. After 24 h, 10 μL of the MTT solution (5 mg/mL) was added to each well in the dark, and the plates were kept for 3 h at 37 °C under a 5% CO_2_ atmosphere. At the end of the incubation, the culture medium was removed, the wells were washed twice with 100 μL of PBS, and the formazan crystals were solubilized with 250 μL of DMSO. Finally, the absorbance was measured at a wavelength of 570 nm using an ELISA plate reader (Bio-Rad, Milano, Italy). The cell viability was expressed as a percentage relative to the untreated cells, cultured in a serum-free medium, set as 100%.

Cell morphology observations were carried out as previously reported [43]. Briefly, the cells were seeded subconfluently onto a 24-well plate, treated with different AFPE concentrations or in the presence of Peaks 1–4, and then observed for 24 h using a phase-contrast Zeiss Axiovert 40 CFL inverted microscope (Carl Zeiss, Milan, Italy) with an LD A-Plan 10×/0.50 Ph2 objective and equipped with a 12.1-megapixel CCD digital capture camera (Canon, PowerShot G9, Cernusco sul Naviglio, Italy). Images were acquired using digital image software (Remote Capture DC, version 2.7.5, Canon, Tokyo, Japan). To avoid serum interference with AFPE or the tested peaks, all of the treatments were performed in serum-free conditions [12,32,44].

## 6. Statistical Analysis

The enzyme assays were performed in triplicate, and the data were expressed as the mean ± SD of at least three independent experiments. For the cell treatments, the statistical significance of the treated samples was determined against control cells (cultured in serum-free medium) by one-way analysis of variance (ANOVA), followed by Bonferroni’s test. Each value represents the mean ± SEM of at least three independent experiments performed in triplicate, using Kaleidagraph (5.04 version) software by Synergy. Significant differences from the untreated control cells were considered as follows: * *p* < 0.05; ^§^ *p* < 0.01; ^#^ *p* < 0.001.

## 7. Results

### 7.1. AFPE Protein Analysis, Biochemical Fractionation, and Identification of ~33 kDa Thaumatin-like Protein 1a

**Figure 1:**
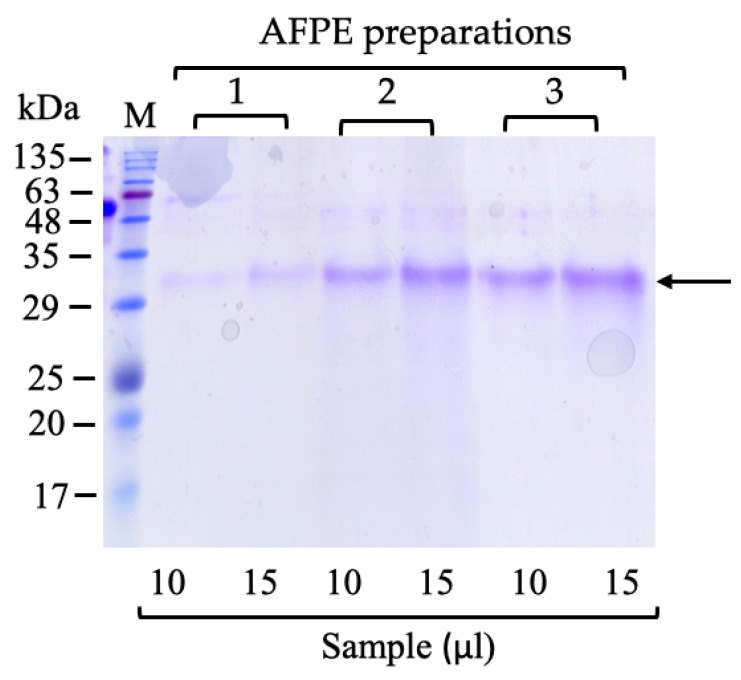
Protein analysis of AFPE by SDS-PAGE: Three different AFPE preparations (1, 2, and 3) were loaded (10 and 15 μL) onto SDS-PAGE, which was carried out using 15% polyacrylamide separating gel, and stained with Coomassie R-250 blue. M, molecular weight marker; the arrow shows the ~33 kDa protein.

To reveal the protein components in AFPE, three different preparations were subjected to a preliminary SDS-PAGE analysis, followed by Coomassie brilliant blue staining (Figure 1). In each preparation, we detected a major protein band with a relative mobility of ~33 kDa. These three different AFPE preparations contained an average ~0.24% protein, as calculated by the comparison between the total protein and PE concentrations (Bradford and Folin assays, respectively) (Table 1).

To identify the ~33 kDa protein, the AFPE was subjected to RP-HPLC analysis, and the eluted fractions were analyzed for protein content by SDS-PAGE (Figure S1a,b). The ~33 kDa protein was separated from the polyphenol fractions by RP-HPLC, and SDS-PAGE analysis showed that it was eluted in Peak 8. The ~33 kDa protein within Peak 8 was identified by high-resolution nano-LC–tandem mass spectrometry (Figure S2). A data-dependent acquisition mode was used, during which higher-energy collisional dissociation (HCD) MS/MS spectra were obtained for the five most intense mass peaks in each scan, allowing for accurate amino acid sequencing of tryptic peptides. Using this approach, six peptide-spectral matches (PSMs) were mapped on the thaumatin-like protein 1 (accession number: Q9FSG7) from *Malus domestica* (Apple). The molecular mass of 25.7 kDa predicted from the deduced amino acid sequence was smaller than that estimated by the relative mobility in the SDS-PAGE (~33 kDa; Figure 1, Figures S1 and S2), as reported for the thaumatin-like (PR5/TL) protein abundantly expressed in apple fruit (*M. domestica* cv. Fuji) [45].

To perform biological studies on the thaumatin-like protein 1a and polyphenol components, AFPE was separated under non-denaturing conditions via gel-filtration chromatography (HiLoad Superdex 75™ 16/60 column).

**Figure 2:**
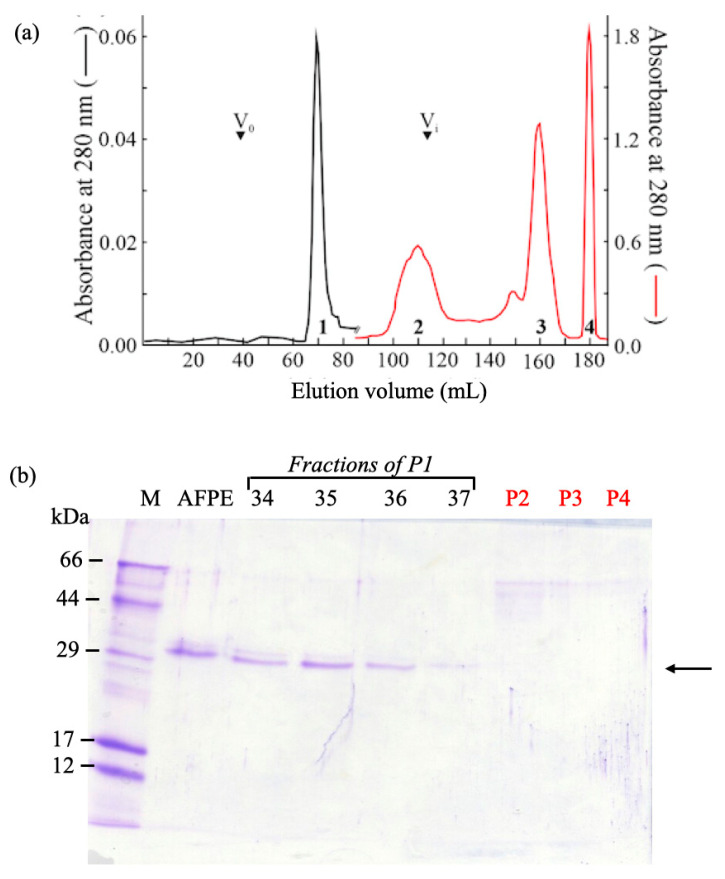
Characterization of AFPE components: (**a**) Elution profile of AFPE components after gel-filtration chromatography (HiLoad Superdex 75™ 16/60 column) monitored at 280 nm. V_0_ and V_i_ indicate the void and inclusion volumes of the gel-filtration column, respectively. (**b**) SDS-PAGE analysis of Peaks 1–4 after gel-filtration separation. Lane 1: M, molecular weight marker; Lane 2: AFPE; Lanes 3, 4, 5, and 6: aliquots of eluted fractions (34, 35, 36, and 37, respectively) from Peak 1. Lanes 7, 8, and 9: aliquots of Peaks 2, 3, and 4, respectively. SDS-PAGE was carried out with a 15% polyacrylamide separating gel and stained with Coomassie blue; the arrow shows the ~33 kDa protein.

The elution profile of the AFPE components after gel filtration (Figure 2a) revealed the presence of at least four peaks, whose elution volume was centered at 70 mL (Peak 1), 110 mL (Peak 2), 160 mL (Peak 3), and 180 mL (Peak 4). Only the components of Peaks 1 and 2 were eluted within the inclusion volume of the column (~120 mL), while the components of Peak 3 had a retarded volume of elution, and the components of Peak 4 were eluted from the column only after the injection of 1.0 M NaOH (1.0 mL). The analysis of the fractions by SDS-PAGE (Figure 2b) revealed that only Peak 1′s fractions contained a protein band with an electrophoretic mobility corresponding to a molecular mass of ~33 kDa, consistent with the gel-filtration elution volume.

### 7.2. Identification of Some Polyphenols in Fractionated AFPE

**Figure 3:**
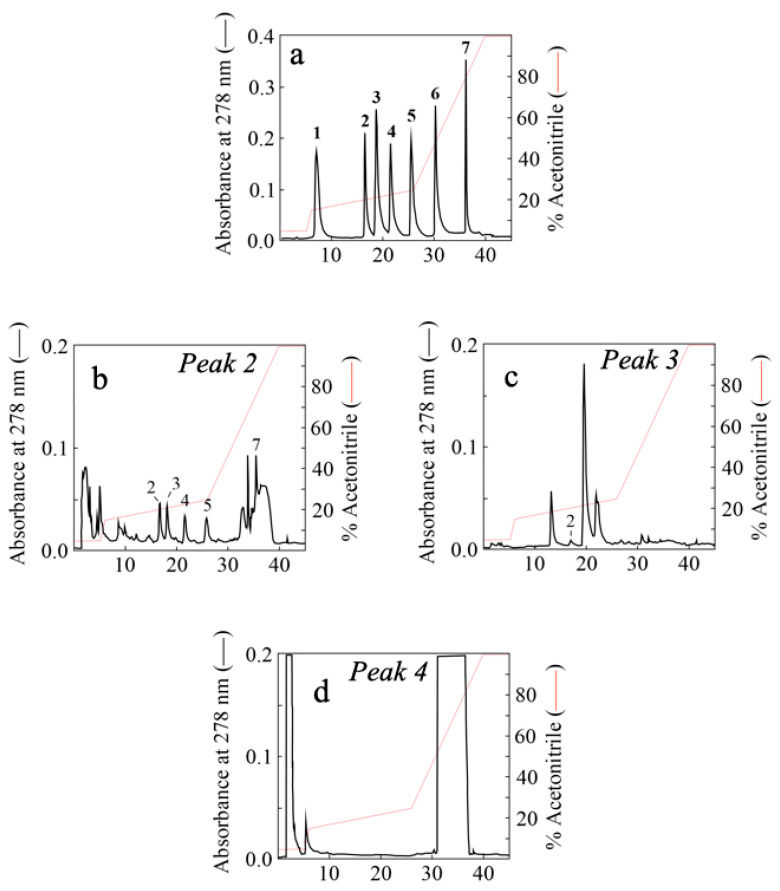
Identification of some polyphenolic components of Peaks 2–4 from fractionated AFPE by RP-HPLC analysis: (**a**) RP-HPLC elution profile of polyphenols used as reference standards: 1, gallic acid (Rt = 6.92; 40 nmol); 2, (+)-catechin (Rt = 16.28; 40 nmol); 3, chlorogenic acid (Rt = 18.48; 40 nmol); 4, caffeic acid (Rt = 21.30; 40 nmol); 5, (–)-epicatechin (Rt = 25.37; 40 nmol); 6, coumaric acid (Rt = 30.12; 40 nmol); 7, quercetin (Rt = 35.03; 40 nmol). (**b**–**d**) RP-HPLC elution profiles of organic compounds from Peaks 2, 3, and 4, respectively.

To qualitatively identify the polyphenolic components of Peaks 2–4 obtained by gel-filtration chromatography of AFPE, an RP-HPLC analysis was carried out (Figure 3). This analysis demonstrated the presence of polyphenols known to be present in *Annurca* apple within our peaks, such as gallic acid, (+)-catechin, chlorogenic acid, caffeic acid, (–)-epicatechin, coumaric acid, and quercetin [32]. In Figure 3a, representative elution profiles of the standard polyphenols are shown, while Figure 3b–d report the elution profiles of the organic compounds retrieved in Peaks 2, 3, and 4, respectively. We found that Peak 2 contained five of the seven representative polyphenols ((+)-catechin, chlorogenic acid, caffeic acid, (–)-epicatechin, and quercetin), while only (+)-catechin was identified in the elution profile of Peak 3. The retention times of peaks within the elution profile of Peak 4 were not matched exactly with those of the available standards, although the more abundant ones appeared close to that observed for quercetin.

### 7.3. Effects of Peaks 1–4 on Cholinesterase Activity

**Figure 4:**
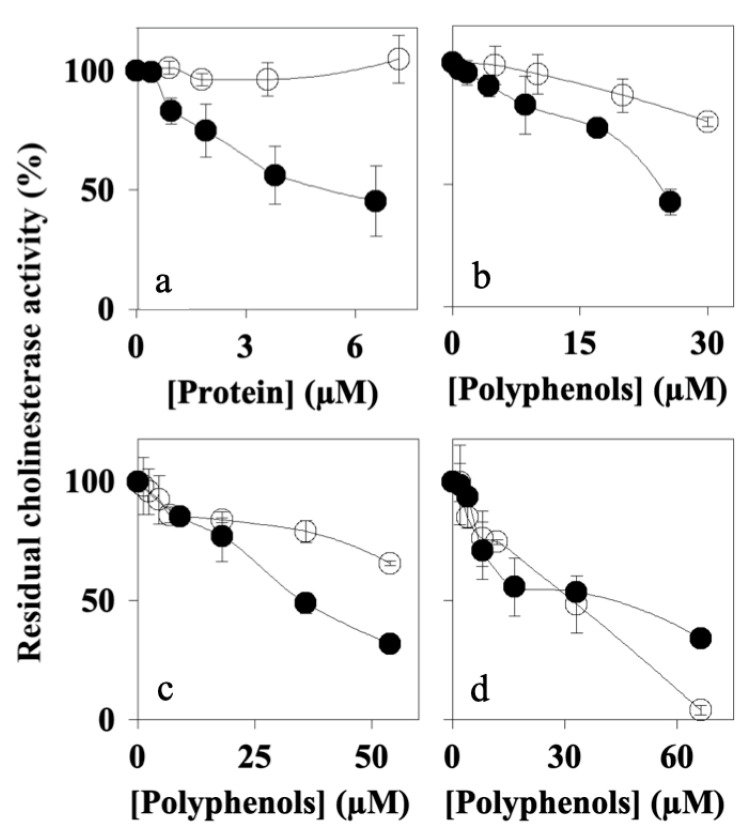
Effects of the peaks obtained by gel filtration on AChE (black circles) or BuChE (open circles) enzyme activity in vitro. The graphs show the residual enzyme activity determined in the presence of the indicated concentrations of protein (Peak 1) or polyphenols (Peaks 2, 3, and 4), as reported in Section 2. (**a**) Peak 1 (thaumatin-like protein 1); (**b**) Peak 2; (**c**) Peak 3; (**d**) Peak 4. The data from semi-logarithmic plots allowed for the calculation of the IC_50_ values that are reported in Table 2.

Since AFPE showed neuroprotective properties [32], we analyzed the ability of Peaks 1–4 to affect the activity of AChE and BuChE, key enzymes involved in AD and PD, using in vitro enzyme assays that were performed in the absence or in the presence of the samples. To determine the IC_50_ values of each peak, we calculated the residual enzyme activity at different concentrations of sample. The results (Figure 4 and Table 2) showed that Peak 1 (thaumatin-like protein 1a; Figure 4a) had a negligible effect on BuChE (IC_50_ not detected), while it was able to inhibit AChE to a greater extent, in a concentration-dependent manner, with an IC_50_ value of 3.53 ± 0.09 μM. Similar behavior was observed for Peak 2 (Figure 4b) and Peak 3 (Figure 4c) (AChE IC_50_ values of 23.43 ± 0.99 μM and 32.30 ± 0.17 μM, respectively), although they also showed a lower inhibition vs. BuChE (IC_50_ values of 73.14 ± 1.29 μM and 101.35 ± 3.6 μM, respectively. Peak 4 (Figure 4d) inhibited both AChE and BuChE (IC_50_ values of 43.67 ± 2.33 μM and 12.70 ± 0.6 μM, respectively) and, at the maximum concentration used, it almost completely abolished BuChE activity.

These results indicated that thaumatin-like protein 1a specifically inhibited AChE; conversely, the polyphenols containing Peaks 2–4, albeit to a different extent, induced decreases in both AChE and BuChE activity.

### 7.4. Effects of AFPE and Peaks 1–4 on Aβ _1–40_ Self-Aggregation and Disaggregation Processes

**Figure 5:**
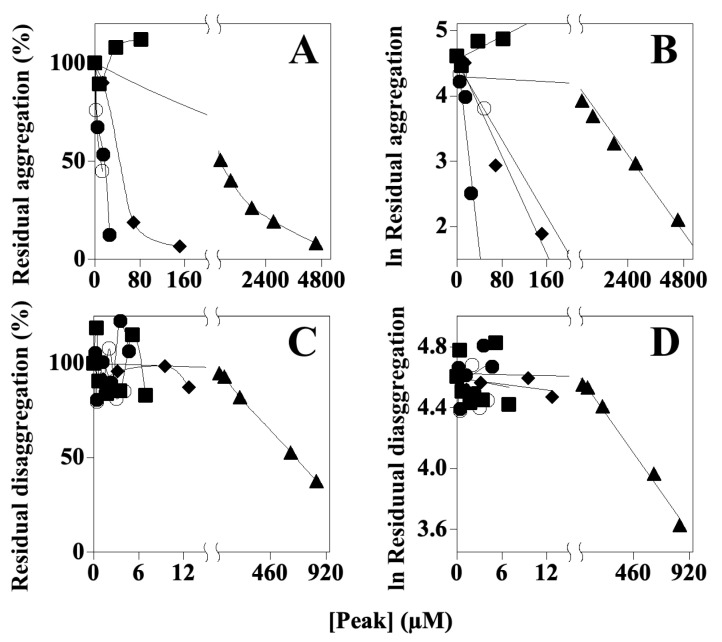
Effects of AFPE and Peaks 1–4 on A*β* _1–40_ self-aggregation and disaggregation processes in vitro: The graphs show the residual fibril aggregation (**A**,**B**) or disaggregation (**C**,**D**) processes, determined in the absence or in the presence of the indicated concentrations of protein for Peak 1 (black circles) or polyphenols for AFPE (black triangles), Peak 2 (empty circles), Peak 3 (black squares), and Peak 4 (black diamonds), as reported in Section 2. Values are expressed as percentages with respect to control samples, in the absence of peak components. The data from semi-logarithmic plots (**b**,**d**) allowed for the calculation of the IC_50_ values that are reported in Table 2.

To evaluate the properties of AFPE and Peaks 1–4 on A*β*_1–40_ amyloidogenesis, we investigated whether these samples might interfere with the A*β* _1–40_ self-aggregation process and/or revert the fibril formation. To this end, the A*β* _1–40_ peptide was incubated alone or in the presence of increasing concentrations of protein (Peak 1) or polyphenols (Peaks 2–4), as reported in Section 2. The results (Figure 5) allowed us to calculate the IC_50_ values, which are summarized in Table 2. The table shows that AFPE inhibited the fibril formation (IC_50_ value: 1396.60 ± 3.05 μM), although it was not able to revert their aggregation (IC_50_ value not detected). Among the various peaks, Peak 1 was the best inhibitor of the aggregation process (IC_50_ value: 9.16 ± 0.52 μM), although it was ineffective in the reversion of fibril aggregation. Peak 2 inhibited fibril aggregation (IC_50_ value: 44.47 ± 1.54 μM); in contrast, Peak 3 did not exert any effect on either process. Notably, Peak 4 exhibited either inhibitory activity against fibril aggregation (IC_50_ value: 37.11 ± 0.41 μM), with better efficacy compared to that of AFPE, or the ability to revert fibril aggregation (IC_50_ value: 104.87 ± 43.02 μM).

### 7.5. Effects of Peaks 1–4 on MAO-A and MAO-B Activity

**Figure 6:**
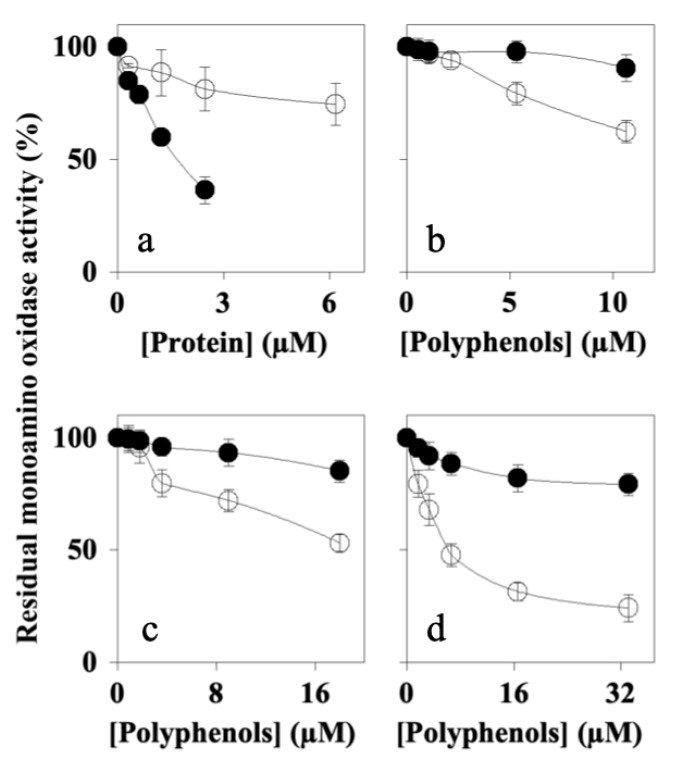
Effects of Peaks 1–4 on MAO-A (black circle) or MAO-B (open circle) activity in vitro: The graphs show the residual enzyme activity determined in the absence or in the presence of the indicated concentrations of protein (Peak 1) or polyphenols (Peaks 2, 3, and 4), as reported in Section 2. (**a**) Peak 1 (thaumatin-like protein 1); (**b**) Peak 2; (**c**) Peak 3; (**d**) Peak 4. The data from semi-logarithmic plots allowed for the calculation of the IC_50_ values that are reported in Table 2.

Since MAO inhibitors are also currently used as drugs for the treatment of neurodegenerative disorders [46,47], next we determined the effects of Peaks 1–4 on MAO-A and MAO-B activity. The results (Figure 6) showed that Peak 1 (Panel a) mostly inhibited MAO-A, with an IC_50_ value (1.71 ± 0.05 μM) lower than that calculated for MAO-B (16.43 ± 1.06 μM). Peaks 2–4 (Panels b, c, and d, respectively) reduced both MAO-A and MAO-B, with a similar effect on MAO-A activity (IC_50_ values between 70 and 85 μM) and a better effect on MAO-B activity (IC_50_ values ranging from 6 to 20 μM). In addition, these results indicate that all of the peaks were able to inhibit MAO-A/B with a better capacity than that of AFPE (IC_50_ values for MAO-A and MAO-B corresponding to 145 and 199 μM, respectively).

These data demonstrate that thaumatin-like protein 1a acted as a selective MAO-A inhibitor, with good efficacy on MAO-A activity compared to Peaks 2–3. In addition, Peak 4 showed a behavior comparable to that of Peak 1.

## 8. Effects of AFPE and Peaks 1–4 on the Cell Viability and Morphology of AGS and MKN-28 Human Gastric Adenocarcinoma Cells and SH-SY5Y Human Neuroblastoma Cells

**Figure 7:**
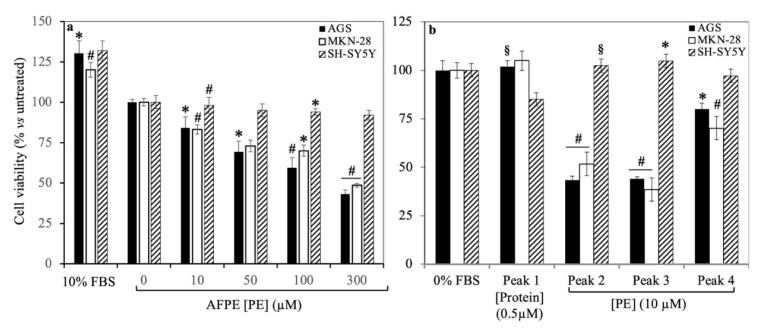
Effects of AFPE and Peaks 1–4 on the viability of AGS, MKN-28, and SH-SY5Y cells: Cells kept in serum-free medium were treated with the indicated amounts of (**a**) AFPE or (**b**) Peaks 1–4 for 24 h and then subjected to MTT assay. The data from semi-logarithmic plots allowed for the calculation of AFPE’s IC_50_ value. For each treatment, the values are expressed as percentages with respect to untreated control cells (set as 100%) kept in serum-free medium. Data represent the mean ± SEM (*n* = 3) of three independent experiments performed in triplicate. The statistical significance of the treated samples compared to control cells (cultured in serum-free medium) was determined by one-way analysis of variance (ANOVA), followed by Bonferroni’s test. Significant differences from the untreated control cells were considered as follows: * *p* < 0.05; ^§^ *p* < 0.01; ^#^ *p* < 0.001.

Since Peaks 1–4 showed potential neuroprotective properties, with a view to their possible use as multitarget agents, we next analyzed their cytotoxicity using in vitro cell models. Therefore, we determined the effects of AFPE and Peaks 1–4 on cell viability in the AGS and MKN-28 human gastric adenocarcinoma cell lines, used as representative cell types of the gastrointestinal system, which were also used to study the anti-inflammatory and chemopreventive properties of lemon peel polyphenol extract [44], and in the SH-SY5Y human neuroblastoma cell line. To this end, we first assessed the IC_50_ values of AFPE by exposing cells to increasing concentrations of the sample (10–300 μM) [32] in serum-free conditions for 24 h. As shown by MTT assays (Figure 7a) in the AGS and MKN-28 cell lines, their viability was inhibited by AFPE in a concentration-dependent manner, and the data analysis using semi-logarithmic plots allowed for the determination of IC_50_ values corresponding to 144 ± 12 μM and 223 ± 7 μM for AGS and MKN-28, respectively. Conversely, the maximal AFPE concentration used (300 μM) only induced a ~20% reduction in the viability of SH-SY5Y cells, in line with previous results [32,48].

Next, the cells were exposed to Peaks 1–4, and the results (Figure 7b) demonstrated that, in AGS and MKN-28 cells, Peak 1 (0.5 μM protein) did not exert a cytotoxic effect, because the cell viability was similar to that observed in untreated cells. Peak 4 (at a 10 μM polyphenol concentration) had little influence on cell viability; conversely, Peaks 2 and 3 (at 10 μM polyphenol concentrations) reduced cell viability to ~60% compared to that of untreated control cells. In SH-SY5Y cells, the exposure to Peaks 1–4 did not affect cell viability as compared with that of untreated cells.

**Figure 8:**
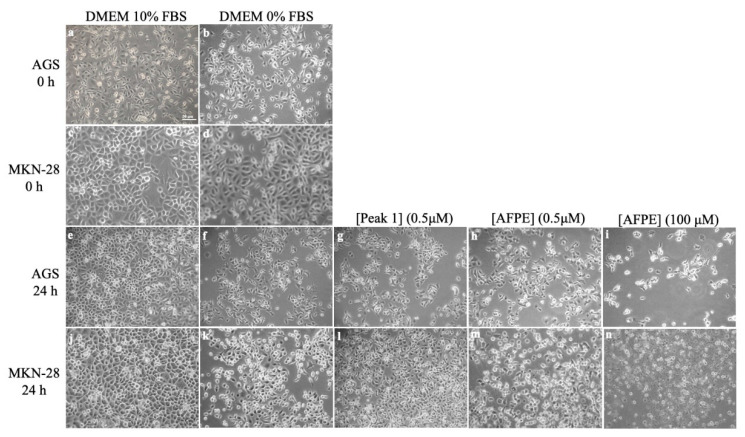
Effects of AFPE and Peak 1 on AGS and MKN-28 cells: Cells kept in serum-free medium were treated for 24 h with the indicated concentrations of protein for Peak 1 or polyphenols for AFPE, and then they were subjected to cell morphology observations. Representative images of AGS and MKN-28 cells were captured at time 0 and 24 h by a phase-contrast microscope (10× objective). Cells kept in complete medium (**a**,**c**,**e**,**j**) or in serum-free conditions (**b**,**d**,**f**,**k**) were treated with the indicated concentrations of Peak 1 (**g**,**l**) or AFPE (**h**,**i**,**m**,**n**). Scale bar = 30 μm.

To confirm the results obtained from the MTT assays, we assessed whether AFPE and Peaks 1–4 could induce morphological changes in the AGS, MKN-28, and SH-SY5Y cells. To this end, the cells were exposed to AFPE or Peaks 1–4 for 24 h and then observed by phase-contrast microscopy. The results obtained for the AGS and MKN-28 cell lines (Figure 8) showed an epithelial-like morphology of exponentially growing cells cultured in complete medium (DMEM 10% FBS) at time 0 (Figure 8a,c) and 24 h (Figure 8e,j). The serum-free conditions, observed in untreated control cells (DMEM 0% FBS) at time 0 (Figure 8b,d), induced an arrest in cell proliferation and rounding of cell bodies at 24 h (Figure 8f,k).

In contrast, in both cell lines, the exposure to AFPE (0.5 μM, Figure 8h,m; 100 μM, Figure 8i,n) caused a strong change in cell morphology; in fact, the cells appeared with round cell bodies and with a lower density compared to the control cells (Figure 8f,k), suggesting a detachment from the surface of the tissue culture dish and/or cell death. This effect was most prominent at the highest concentration of AFPE (100 μM, Figure 8i,n).

It should be noted that, in both cell lines, the treatments with Peak 1 (0.5 μM protein, Figure 8g,l) did not alter the cell morphology, which was similar to that of untreated cells kept in serum-free medium at 24 h (Figure 8f,k). In addition, the treatment with Peak 1 protected the cells from the rounding and decreased their adherence to the culture dish induced by serum deprivation conditions (compare Figure 8g,l with Figure 8f,k).

**Figure 9:**
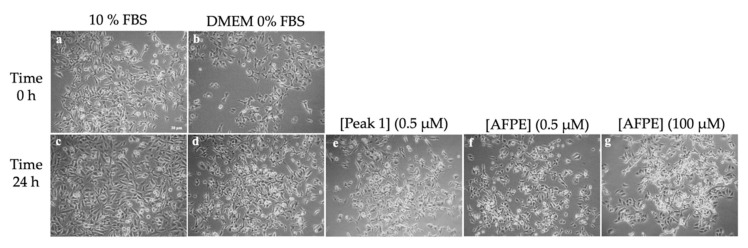
Effects of AFPE and Peak 1 on SH-SY5Y cells: Cells kept in serum-free medium were treated for 24 h with the indicated concentrations of protein for Peak 1 or polyphenols for AFPE, and then they were subjected to cell morphology observations. Representative images of cells captured at time 0 and 24 h by phase-contrast microscopy (10× objective). Cells kept in complete medium (**a**,**c**) or in serum-free conditions (**b**,**d**) were treated with the indicated concentrations of Peak 1 (**e**) or AFPE (**f**,**g**). Scale bar = 30 μm.

In the SH-SY5Y neuroblastoma cells (Figure 9), we did not observe relevant changes in cell morphology in the comparison between the treated (Figure 9e–g) and control cells (Figure 9d).

Finally, we determined the effect of Peaks 2–4 on the morphology of AGS, MKN-28, and SH-SY5Y cells (Figures S4 and S5). In AGS and MKN-28 cells, the treatments with Peaks 2 and 3 (10 μM, for 24 h) induced effects similar to those exerted by AFPE, i.e., a reduced cell number with concomitant cell rounding; conversely, the exposure to Peak 4 (10 μM, for 24 h) did not alter the cell density and morphology, which were similar to those of untreated cells kept in serum-free medium. In SH-SY5Y cells, no significant effects were observed.

These results indicate that, under our experimental conditions and at the tested concentrations, cytotoxic effects and changes in cell morphology were detected only in AGS and MKN-28 cells treated with AFPE or Peaks 2–4. In these cells, Peak 1 (thaumatin-like protein 1a) did not induce cytotoxic effects; rather, it acted as a protective factor, counteracting the detachment caused by serum deprivation conditions.

## 9. Discussion

In agreement with recent evidence reporting that plant proteins can also be considered for their therapeutic potential and neuroprotective functions [49,50,51], our results demonstrated the presence of a protein component in AFPE, as revealed by the biochemical purification of the extract. A preliminary analysis of the total protein content of AFPE by SDS-PAGE (Figure 1) demonstrated the presence of an abundant protein with a relative mobility corresponding to ~33 kDa. This result is consistent with previous evidence demonstrating high amounts of a protein with similar mobility in various *Annurca* apple cultivars from the south of Italy [14,19].

Here, we report the purification to apparent homogeneity of thaumatin-like protein 1a from AFPE. The protein isolation and the search of the PDB for its tryptic peptides (Figures S1 and S2) revealed its identity as thaumatin-like protein 1a, gene *TL1* from *Malus domestica* (apple) (*Pyrus malus*) (UniProt accession number: Q9FSG7). Concerning its molecular mass, we observed a discrepancy between that determined for the purified protein with relative mobility of ~33 kDa on SDS-PAGE (Figure 1) and that predicted based on the amino acid sequence (25.7 kDa). This difference could be explained by the occurrence of protein glycosylation, as in the case of the thaumatin-like (PR5/TL) protein abundantly expressed in apple fruit (*Malus domestica* cv. Fuji) [45].

To perform biological studies, AFPE was fractionated under non-denaturing conditions by gel-filtration chromatography (Figure 2a), and after SDS-PAGE analysis (Figure 2b) the fractions containing thaumatin-like protein 1a (Peak 1) or polyphenol components (Peaks 2–4) were pooled together for further analysis.

Several scientific works have reported that apple-derived polyphenols are a rich source of nutraceutical biomolecules with beneficial effects on human health. Most of these compounds are polyphenols with antioxidant activity [14,52,53,54], while some of them also exhibit antiproliferative action [33,53] and can modulate some of the key enzymes implicated in oxidative stress [32]. In this framework, the neuroprotective properties of the polyphenol-containing Peaks 2–4 (Figure 4, Figure 5 and Figure 6) may be correlated with the most represented polyphenol components, such as gallic acid, (+)-catechin, chlorogenic acid, caffeic acid, (–)-epicatechin, coumaric acid, and quercetin (Figure 3). These results are consistent with those previously obtained for AFPE and silymarin, a plant-derived flavonoid [32,33], and further allowed us to determine the IC_50_ values of thaumatin-like protein 1a and the polyphenol-containing Peaks 2–4.

The enzyme-inhibitory properties and the ability to affect the A*β* _1–40_ peptide aggregation process of Peaks 1–4 were explored using in vitro assays that allowed for direct and specific molecular interactions between the AFPE components and the target domain(s) involved in either cholinesterase and MAO’s enzyme catalytic activity or intermolecular sites triggering fibrils’ aggregation and/or reversion. These results demonstrated that, although to a different extent and specificity, each peak exerted better neuroprotective activity than the non-fractionated AFPE. In fact, as reported in Table 2, all of the components of Peaks 1–4 showed IC_50_ values significantly lower than those demonstrated by AFPE for cholinesterase and monoamine oxidases [32]. These results suggest a possible negative interaction among the various components or the presence of molecules decreasing the enzyme-inhibitory activity in AFPE.

In the multitarget therapeutic approach for AD and PD, the identification of molecules either acting as enzyme inhibitors or preventing A*β* amyloidogenesis represents a promising strategy. Our data demonstrated that although Peaks 2 and 3 were ineffective against the fibrillation process, Peak 4, showing anti-cholinesterase and anti-MAO-A/B activity, reduced the aggregation of the fibrils, and this was the only peak capable of reversing their formation even better that the non-fractionated AFPE.

In our experimental model, thaumatin-like protein 1a (Peak 1) clearly emerged as the best inhibitor of AChE, A*β* _1–40_ self-aggregation, and MAO-A compared with the AFPE peaks and total AFPE; therefore, this protein behaved as a multitarget ligand [55].

This observation is consistent with the results of previous studies reporting the ability of osmotin, a PR-5 protein, to provide neuroprotective effects in both in vivo and in vitro models of AD and PD [27,28,29,30,56].

In addition, this neuroprotective behavior is corroborated by the finding that the amino acid sequence alignment between osmotin (accession number Q40529) and thaumatin-like protein 1a (accession number Q9FSG7) shows 39% and 54% sequence identity and similarity, respectively (Figure S3), making a structure–function relationship between osmotin and thaumatin-like protein 1a purified from AFPE likely.

Finally, we analyzed the effects of Peaks 1–4 on cell viability and morphology in AGS and MKN-28 human gastric adenocarcinoma cells and SH-SY5Y human neuroblastoma cells (Figure 7, Figure 8 and Figure 9, Figures S4 and S5). Considering the overall results obtained under our experimental conditions for cell viability and morphology in the tested cell lines, it clearly emerged that AGS and MKN-28 cells showed a greater sensitivity to the cytotoxic effects induced by AFPE and Peaks 1–4 compared to that shown by SH-SY5Ycells. Regarding AGS and MKN-28 cells, they were exposed to AFPE or Peaks 1–4 at concentrations lower than those corresponding to the IC_50_ values of AFPE for AGS and MKN-28 cells (114 ± 12 µM and 223 ± 7 µM, respectively) in serum-free culture medium, to avoid interactions among the FBS components and the tested samples [32,44]. We observed that in these cells, the treatment with Peaks 2–4 greatly reduced the cell viability. Conversely, the exposure to Peak 1 did not affect either cell viability or cell morphology, preventing the cell rounding and detachment caused by serum deprivation. The most intriguing finding of our study is the first demonstration of the protective behavior exerted by thaumatin-like protein 1a on AGS and MKN-28 cells. This effect could be due to a possible interaction of the protein with plasma membrane components that protected the cells from the serum deprivation, thus simulating stress conditions. In light of this observation, since various human gastric cancer cell lines, including MKN-28 cells, express both AdipoR1 and AdipoR2 receptors [57], we might presume a possible interaction between thaumatin-like protein 1a and the adiponectin receptor(s), as previously demonstrated for osmotin [25].

The similar neuroprotective effect of thaumatin-like protein 1a to that of the other food protein osmotin, both belonging to the PR-5 family, suggests a cross-reactivity between these members, although further studies will be required.

Although these results clearly demonstrate the neuroprotective functions exerted by thaumatin-like protein 1a and the other peak components purified from AFPE, we must consider some limitations depending on the experimental models used. We assessed the ability of these samples to inhibit cholinesterase and monoamine oxidase activity, along with their effects on the A*β* _1–40_ aggregation/reversion process, using in vitro assays that did not mimic the cell context, in the absence of its receptors, metabolism, and bio-signaling pathways. However, these results represent a good starting point for further studies using in vitro cellular models of AD and PD.

Regarding the biological roles of thaumatin-like protein 1a identified in *Malus* × domestica Borkh. cv. *Annurca*, previous studies have demonstrated that this protein plays different biological functions, such as enzymatic, structural, and defense functions; however, it is mostly involved in apple allergenicity [14,15,19]. Hence, in this study, we demonstrated for the first time that thaumatin-like protein 1a isolated from AFPE acted in a pleiotropic manner, behaving like a multitarget ligand. In fact, it inhibited AChE, MAO-A, and A*β* _1–40_ fibril aggregation and protected AGS and MKN-28 cells against stress conditions induced by serum deprivation.

Our data suggest the potential use of these AFPE components in the prevention and amelioration of the symptoms of neurodegenerative disorders such as AD and PD.

## 10. Conclusions

Taken together, our results demonstrate the purification and the novel neuroprotective activity of thaumatin-like protein 1a from AFPE. This protein was able to inhibit key enzymes and A*β* _1–40_ aggregation involved in the neuronal death and neurodegenerative processes that occur in AD and PD.

Furthermore, the results reported here clearly indicate that the best and most differential effects can be associated with the different components of the AFPE, in comparison with the total extract. In fact, the findings reported here, in addition to providing new insights into the biological function of thaumatin-like protein 1a from *Annurca* apple, also indicate that AFPE’s effects cannot be attributed only to its polyphenol components, but also to thaumatin-like protein 1a. In addition, a specific effect on the A*β* fibril disaggregation can be ascribed to the polyphenolic components (mainly quercetin) contained in Peak 4. These results will contribute to the development of novel diet-based/plant factors for potential strategies aiming at preventing and ameliorating the symptoms of neurodegenerative disorders.

## Figures and Tables

**Table 1:** Concentrations of total proteins and polyphenols in three different AFPE preparations, and total protein percentages with respect to polyphenol contents.

AFPE Preparations	Proteins (mM)	Polyphenols (mM)	Proteins/Polyphenols (%)
1	0.017	10.00	0.17
2	0.029	8.60	0.34
3	0.033	15.00	0.22
Mean ± S.D.	0.026 ± 0.008	11.20 ± 3.40	0.24 ± 0.09

**Table 2:** IC_50_ values (µM) ± SD determined vs. protein or polyphenol (PE) concentrations of AFPE and Peaks 1–4 for the inhibition of enzymes, as well as A*β* _1–40_ fibril formation and reversion.

	AFPE	Peak 1	Peak 2	Peak 3	Peak 4
Enzyme	PE (µM)	Protein (µM)	PE (µM)	PE (µM)	PE (µM)
AChE	859 ± 18 ^#^	5.53 ± 0.09	23.43 ± 0.99	32.30 ± 0.17	43.67 ± 2.33
BuChE	966 ± 72 ^#^	N.D.	73.14 ± 1.29	101.35 ± 3.60	12.70 ± 0.60
MAO-A	145 ± 30 ^#^	1.71 ± 0.05	84.61 ± 5.64	79.60 ± 0.50	62.92 ± 2.48
MAO-B	199 ± 70 ^#^	16.43 ± 1.06	15.77 ± 0.12	19.63 ± 0.34	6.33 ± 0.02
Fibrillation process					
Inhibition of A*β* _1–40_ fibril formation	1396.00 ± 3.05	9.16 ± 0.52	44.47 ± 1.54	N.D.	37.11 ± 0.41
Reversion of A*β* _1–40_ fibrils	616.70 ± 2.65	N.D.	N.D.	N.D.	104.87 ± 43.02

Values are expressed as the mean ± SD calculated from at least three different determinations; ^#^ reference [32].

## Data Availability

Data are contained within the article and Supplementary Materials.

## Supplementary Materials

The following supporting information can be downloaded at: https://www.mdpi.com/article/10.3390/nu16020307/s1, Figure S1: (a) Elution profile of RP-HPLC analysis of AFPE (50 μL) on a C-4 column (250 mm × 4.6 mm, 5 μm particle size; 300 Å; Phenomenex, Castel Maggiore, Bologna, Italy); (b) 15% SDS-PAGE of RP-HPLC analysis of Peaks 1–8 and the total AFPE extract (10 μL). Figure S2: Amino acid sequences of tryptic peptides mapped on thaumatin-like protein 1a, identified by high-resolution nano-LC–tandem mass spectrometry (AC: Q9FSG7). Within the table, sequences, numbers of missed cleavage sites (MC), experimental masses of precursor ions, charge states, and the experimental and theoretical molecular weights of peptides (MH+), together with mass accuracies and retention times, are reported; c = carbamidomethyl cysteine. Figure S3: Pairwise protein sequence alignment between osmotin (accession number Q40529) and thaumatin-like protein 1a (accession number Q9FSG7) was performed by querying the RCSB PDB (Protein Data Bank) (https://www.rcsb.org/alignment, accessed on 30 November 2023). Figure S4: Effects of Peaks 2–4 on AGS and MKN-28 cells. Cells kept in serum-free medium were treated with the indicated concentration of each sample for 24 h and then subjected to cell morphology analysis. Representative images of AGS and MKN-28 cells were captured at time 0 and 24 h by a phase-contrast microscope (10× objective). Cells kept in complete medium (DMEM 10% FBS) or in serum-free conditions (DMEM 0% FBS) were treated with the indicated concentration of each peak. Figure S5: Effects of Peaks 2–4 on SH-SY5Y cells’ morphology. Cells plated onto 96-well plates were exposed to the indicated concentration of each sample for 24 h and then subjected to cell morphology observation. Representative images of cells were captured at time 0 and 24 h by phase-contrast microscopy (10× objective).
