# Supplementary material for: Identification and Characterization of Neuroprotective Properties of Thaumatin-like Protein 1a from Annurca Apple Flesh Polyphenol Extract
Source: Nutrients. 2024 Jan 19;16(2):307. doi: 10.3390/nu16020307 (PMC10821328; doi:10.3390/nu16020307)
Supplement: Supplementary file 1 [file nutrients-16-00307-s001.zip › nutrients-2799651-supplementary.pdf]

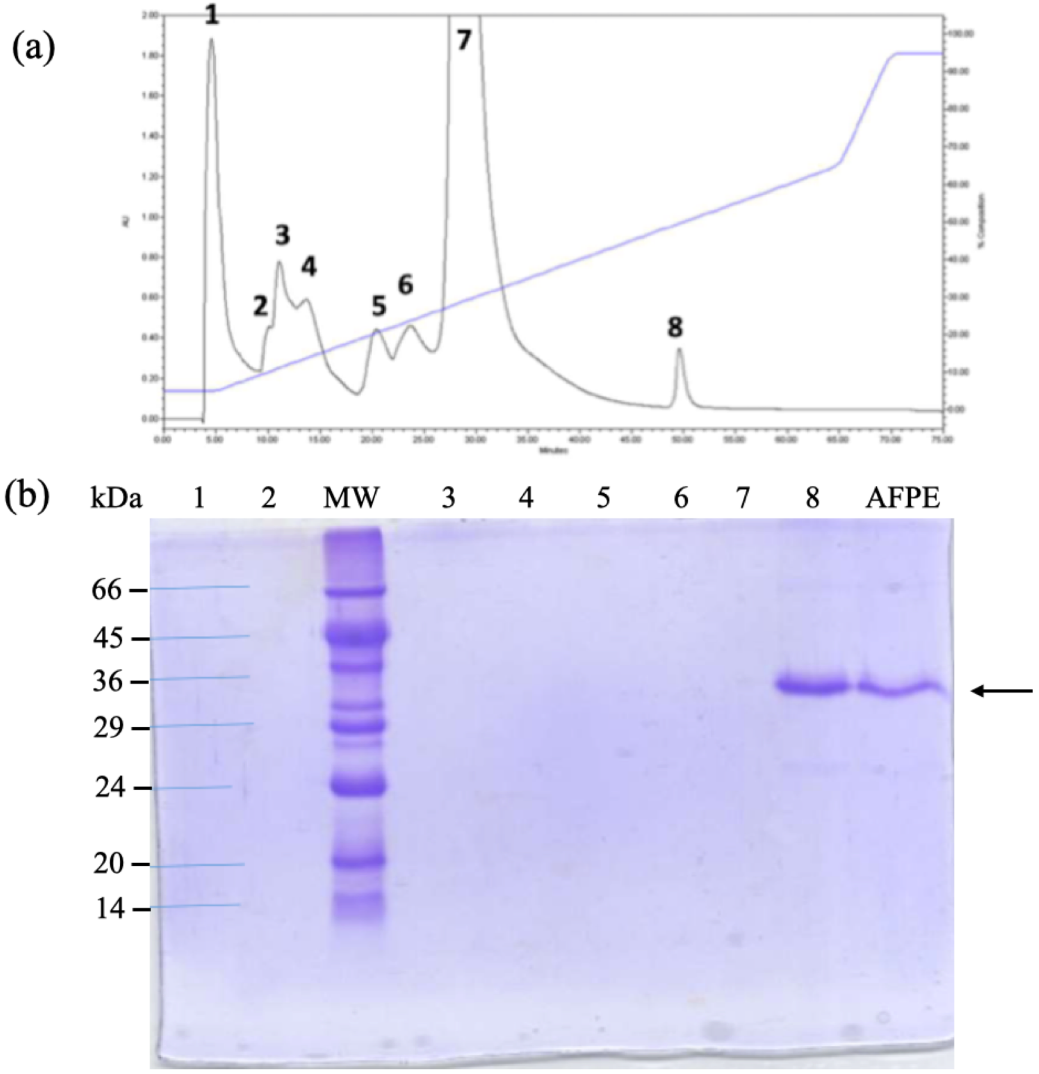

Figure S1: (**a**) Elution profile of RP-HPLC analysis of AFPE (50 µl) on a C-4 column (250 mm × 4.6 mm, 5-µm particle size; 300 Å; Phenomenex, Castel Maggiore, Bologna, Italy); (**b**) 15% SDS-PAGE of RP-HPLC analysis of Peaks 1 - 8 and AFPE total extract (10 µl). Arrow shows the 33 kDa protein.

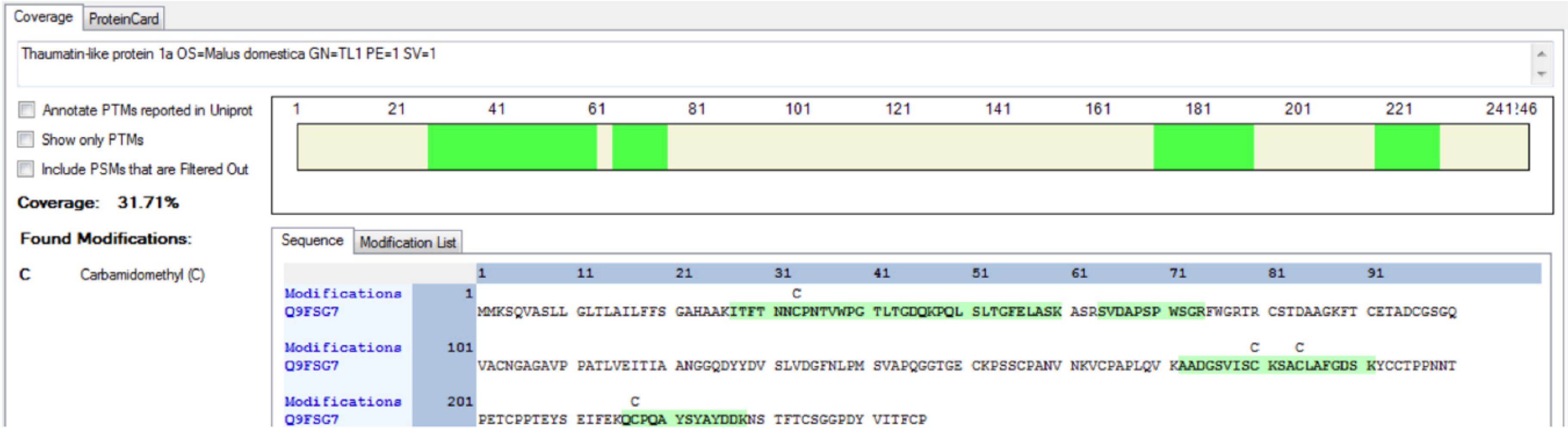

Accession	Description	Coverage	# Peptides	# PSMs	# Unique Peptides	# AAs	MW [kDa]	calc. pI	Score Sequest HT	# Peptides Sequest HT
Q9FSG7	Thaumatin-like protein 1a OS=Malus domestica GN=TL1 PE=1 SV=1	31	5	6	5	246	25,683	5,15	12,0395534	5

Annotated Sequence	Modifications	# Protein Groups	# Proteins	Master Protein Accessions	# Missed Cleavages	Charge	m/z [Da]	MH+ [Da]	DeltaM [ppm]	RT [min]
[K].QcPQAYSYAYDDK.[N]	C2(Carbamidomethyl)	1	2	Q9FSG7	0	2	804,8347	1608,662	-0,83398	12,57942
[K].AADGSVIScK.[S]	C9(Carbamidomethyl)	1	2	Q9FSG7	0	2	504,2451	1007,483	0,394488	11,09707
[K].SAcLAFGDSK.[Y]	C3(Carbamidomethyl)	1	2	Q9FSG7	0	2	528,2454	1055,483	0,781336	12,91876
[K].AADGSVIScK.[S]	C9(Carbamidomethyl)	1	2	Q9FSG7	0	2	504,2448	1007,482	-0,21133	11,25774
[R].SVDAPSPWSGR.[F]		1	2	Q9FSG7	0	2	579,781	1158,555	0,79669	13,25838
[K].ITFTNNcPNTVWPGTLTGDQKPQLSLTGFELASK.[A]	C7(Carbamidomethyl)	1	1	Q9FSG7	0	4	934,7219	3735,866	-0,82041	16,42328

Figure S2: Amino acid sequences of tryptic peptides mapped on Thaumatin-like protein 1a identified by high-resolution nanoLC-Tandem Mass Spectrometry (AC: Q9FSG7). Within the Table, sequences, number of missed cleavages sites (MC), experimental masses of precursor ions, charge state, experimental and theoretical molecular weights of peptides (MH+) together with mass accuracies and retention times are reported. c = carbamidomethyl cysteine.

```
>sp|Q9FSG7|TP1A_MALDO Thaumatin-like protein 1a OS=Malus domestica OX=3750 GN=TL1 PE=1 SV=1
Sequence ID: Query_8427 Length: 246
Range 1: 2 to 246

Score:157 bits(398), Expect:2e-52,
Method:Compositional matrix adjust.,
Identities:97/249(39%), Positives:136/249(54%), Gaps:27/249(10%)

Query  1    MGNLRSSFVFFLLALVTYT--YAATIEVRNNCPYTVWAASTPIGGGRRLDRGQTWVINAP  58
            M +  +S +   LA++ ++  +AA I   NNCP TVW  +  + G ++     T    A
Sbjct  2    MKSQVASLLGLTLAILFFSGAHAAKITFTNNCPNTVWPGT--LTGDQKPQLSLTGFELAS  59

Query  59   RGTKMA--------RVWGRTNCNFNAAGRGTCQTGDCG-GVLQCTGWGK-PPNTLAEYAL  108
            + ++          R WGRT C+ +AAG+ TC+T DCG G + C G G  PP TL E  +
Sbjct  60   KASRSVDAPSPWSGRFWGRTRCSTDAAGKFTCETADCGSGQVACNGAGAVPPATLVEITI  119

Query  109  DQFSGLDFWDISLVDGFNIPMTFAPTNPSGGKCHAIHCTANINGECPRELRVPGG-----  163
                G D++D+SLVDGFN+PM+ AP   +G +C    C AN+N  CP  L+V
Sbjct  120  AANGGQDYYDVSLVDGFNLPMSVAPQGGTG-ECKPSSCPANVNKVCPAPLQVKAADGSVI  178

Query  164  -CNNPCTTFGGQQYCCTQ-----GPCGPTFFSKFFKQRCPDAYSYPQDDPTSTFTCPGGS  217
             C + C  FG  +YCCT        C PT +S+ F+++CP AYSY  DD  STFTC GG
Sbjct  179  SCKSACLAFGDSKYCCTPPNNTPETCPPTEYSEIFEKQCPQAYSYAYDDKNSTFTCSGGP  238

Query  218  TNYRVIFCP  226
             +Y + FCP
Sbjct  239  -DYVITFCP  246
```

Figure S3: Pairwise protein sequence alignment between Osmotin (accession number Q40529) and Thaumatin-like 1a protein (accession number Q9FSG7) was performed by query on RCSB PDB Protein Data Bank.

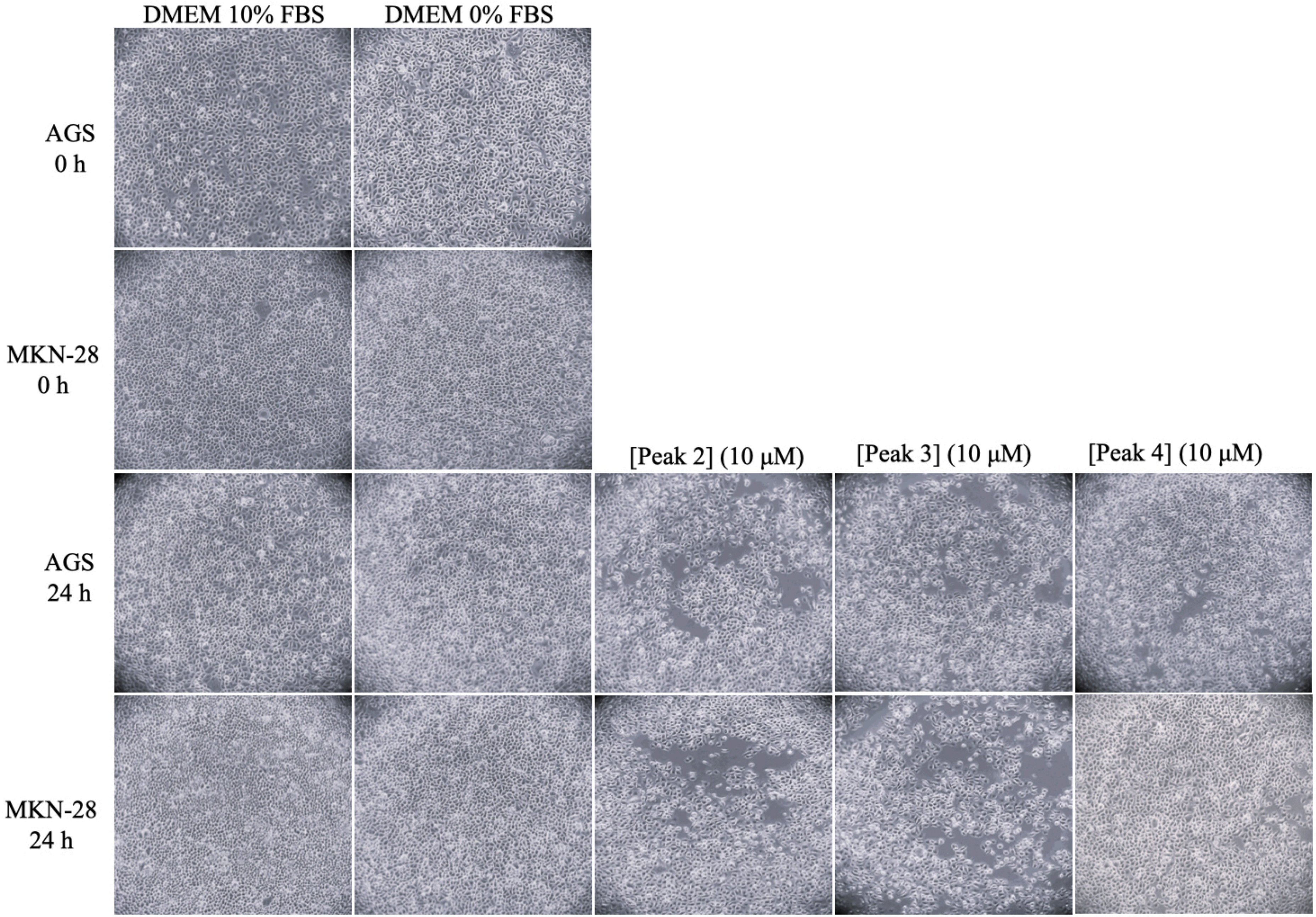

Figure S4: Effects of Peaks 2-4 on AGS and MKN-28 cell morphology. Cells kept in serum-free medium were treated with the indicated concentration of each sample, for 24 h and then subjected to cell morphology analysis. Representative images of AGS and MKN-28 cells captured at time 0 and 24 h by a phase-contrast microscope (10 × objective). Cells kept in serum-free conditions (DMEM 0% FBS) were treated with the indicated concentration of each Peak.

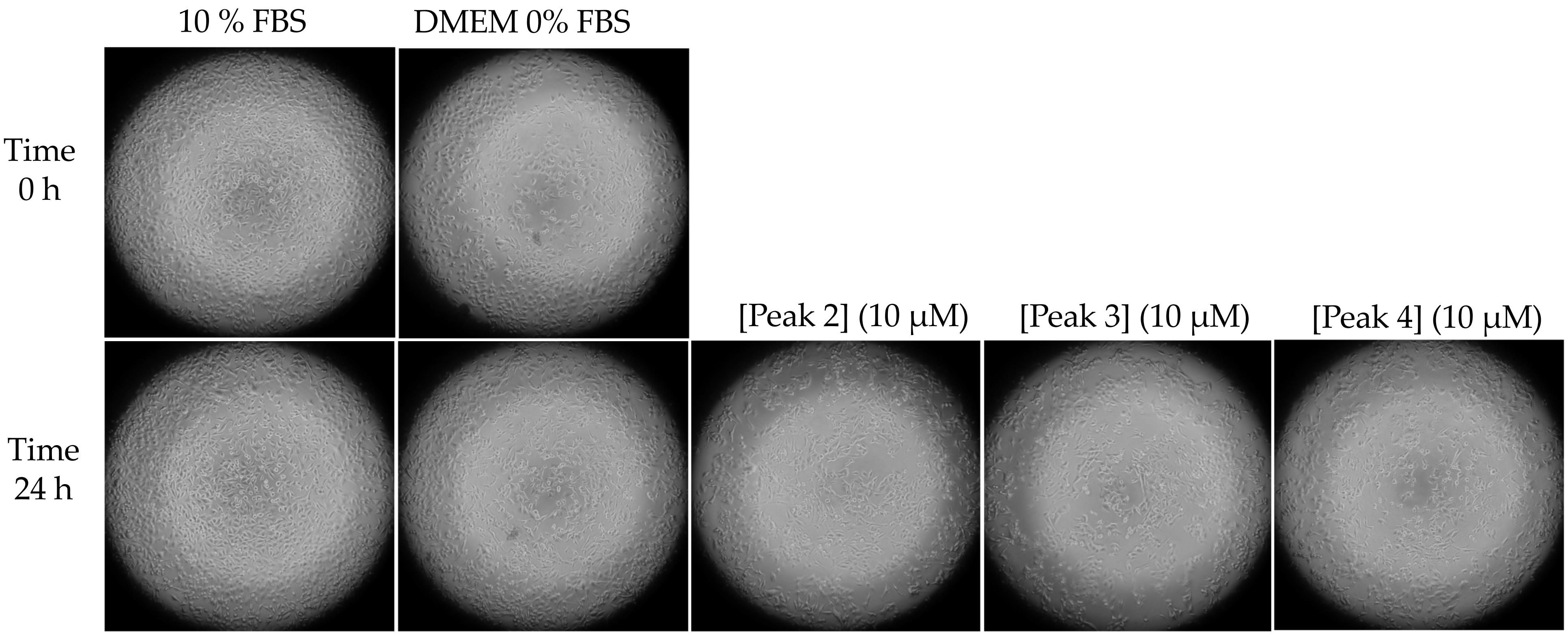

Figure S5: Effects of Peaks 2-4 on SH-SY5Y cell morphology. Cells plated onto 96-well plate were exposed to the indicated concentration of each sample, for 24 h and then subjected to cell morphology obervation. Representative images of cells captured at time 0 and 24 h by phase-contrast microscopy (10 × objective).
